# Impact of nutritional index on contrast-associated acute kidney injury and mortality after percutaneous coronary intervention

**DOI:** 10.1038/s41598-021-86680-7

**Published:** 2021-03-29

**Authors:** Miyeun Han, Hye Won Lee, Han Cheol Lee, Hyo Jin Kim, Eun Young Seong, Sang Heon Song

**Affiliations:** 1 Division of Nephrology, Department of Internal Medicine, Pusan National University Hospital, Busan, Korea; 2 Division of Cardiology, Department of Internal Medicine, Pusan National University Hospital, Busan, Korea; 3 Biomedical Research Institute, Pusan National University Hospital, Busan, Korea; 4 Present Address: Division of Nephrology, Department of Internal Medicine, Hallym University Hangang Sacred Heart Hospital, Seoul, Korea

**Keywords:** Interventional cardiology, Nephrology

## Abstract

The risk of malnutrition in acute kidney injury and mortality in coronary artery disease patients has not been studied. This study aimed to evaluate whether nutritional status assessed by Onodera’s prognostic nutritional index (PNI) was related to percutaneous coronary intervention (PCI) outcomes. A total of 3731 patients who received PCI between January 2010 and December 2018 were included. The relationship between PNI at the time of PCI and the occurrence of contrast-associated acute kidney injury (AKI) and all-cause death was evaluated using logistic regression and Cox proportional hazards models, respectively. AKI occurred in 271 patients (7.3%). A low PNI was independently associated with an increased risk of AKI on multivariate logistic regression analysis (OR 0.96, 95% CI 0.94–0.98, *P* = 0.001). During the median follow-up of 4.3 years, Kaplan–Meier analysis showed that patients with AKI/low PNI < 47.8 had a higher death rate. After adjusting for various risk factors, a low PNI was a significant risk factor for mortality (HR 0.98, CI 0.96–0.99, *P* = 0.003). A low level of PNI was associated with increased mortality, especially in the group aged over 70 years and female sex. PNI was closely associated with acute kidney outcomes and patient mortality after PCI.

## Introduction

Coronary artery disease is the leading cause of morbidity and premature mortality worldwide^1^. Percutaneous coronary intervention (PCI) is a non-surgical revascularization procedure. PCI relieves symptoms of ischemia and offers survival benefits in ischemic heart disease patients^2^. However, the administration of contrast media during angioplasty can result in contrast-associated acute kidney injury (CA-AKI). This complication is associated with increased intra-hospital stay, long-term decline in kidney function, and increased mortality^3,4^. Although there are many differences depending on the study, the incidence of CA-AKI can occur in as much as 1–50%^5,6^. Several risk factors, such as preexisting chronic kidney disease (CKD), old age, hemodynamic instability, osmolality and volume of contrast media, have been established, and several risk stratification models to predict CA-AKI using various factors have been developed in previous studies^6,7^.

Malnutrition is highly prevalent among AKI patients^8,9^, and poor nutritional status could increase the incidence of AKI^10^. Nutrition is associated with immune dysfunction and inflammatory processes^10,11^. AKI, known to be systemic inflammation causing immune cell dysfunction and altered cytokine homeostasis^11^, is also affected by malnutrition. Furthermore, nutrition and diet play an important role in preventing and progressing cardiovascular disease^12^, and malnutrition is a poor prognostic factor for heart disease. However, the effect of undernutrition on CA-AKI and outcome after PCI has not been actively studied.

There are various nutritional assessment tools. The prognostic nutritional index (PNI), calculated from one’s serum albumin concentration and total lymphocyte count in the peripheral blood, was developed by Onodera^13^ to determine the nutritional and inflammatory status of surgical patients. PNI is a simple indicator used not only in postoperative outcomes in patients but also in evaluating the nutritional status of patients with malignancies^14,15^, pulmonary^16,17^, heart^18,19^, and kidney diseases^20^. However, the prognostic value of PNI in CA-AKI and patient mortality after PCI have not yet been validated. We hypothesized that nutritional status was closely related to the occurrence of AKI and patient prognosis after PCI, independent of other well-known risk factors. This study aimed to investigate whether nutritional status assessed by PNI contributed to acute kidney outcomes and patient mortality after PCI.

## Results

### Clinical characteristics of subjects with contrast-associated acute kidney injury

The mean age was 65.4 ± 11.3 years, and 1060 (28.4%) were female. A total of 1061 (28.4%) patients had diabetes mellitus, and 1610 (43.2%) had hypertension. Acute kidney injury (AKI) occurred in 271 patients (7.3%). Table 1 shows the clinical characteristics between the no AKI and AKI groups. Patients with AKI were older and had a higher proportion of females, diabetes mellitus, and hypertension. Body mass index was lower in the AKI group. The levels of hemoglobin, albumin, total lymphocyte count, and eGFR were significantly lower in the AKI group. In contrast, the levels of red cell distribution width coefficient of variation (RDW-CV), creatinine, C-reactive protein (CRP), and uric acid were significantly higher in the AKI group. The proportion of patients with proteinuria was higher in the AKI group. The mean PNI level was 52.5 in the no AKI group and 46.6 in the AKI group. The volume of injected contrast media was higher in the AKI group.

**Table 1:** Clinical characteristics of subjects between no AKI and AKI.

Variables	Total (n = 3731)	No AKI (n = 3460)	AKI (n = 271)	*p* value
Age, years	65.4 ± 11.3	65.1 ± 11.2	69.0 ± 10.8	< 0.001
Sex (female), n (%)	1060 (28.4)	964 (27.9)	96 (35.4)	0.010
Diabetes mellitus, n (%)	1061 (28.4)	935 (27.0)	126 (46.5)	< 0.001
Hypertension, n (%)	1610 (43.2)	1459 (42.2)	151 (55.7)	< 0.001
Current smoker, n (%)	1212 (32.5)	1132 (32.8)	80 (29.6)	0.322
Systolic BP, mmHg	127.6 ± 20.4	127.5 ± 19.9	129.0 ± 25.3	0.348
Diastolic BP, mmHg	76.2 ± 13.2	76.1 ± 13.0	77.1 ± 15.8	0.328
Body mass index, kg/m^2^	24.3 ± 3.2	24.3 ± 3.2	23.7 ± 3.6	0.014
Hemoglobin, g/dL	13.0 ± 2.0	13.1 ± 1.9	11.9 ± 2.3	< 0.001
RDW-CV, %	13.3 ± 1.3	13.3 ± 1.3	13.7 ± 1.3	< 0.001
Total lymphocytes, count/μL	2049.4 ± 1009.6	2066.8 ± 992.4	1827.0 ± 1188.0	0.001
Albumin, g/dL	4.2 ± 0.50	4.2 ± 0.5	3.8 ± 0.6	< 0.001
Creatinine, mg/dL	1.01 ± 0.63	0.97 ± 0.55	1.62 ± 1.14	< 0.001
eGFR, mL/min/1.73m^2^	79.5 ± 23.2	81.4 ± 21.2	56.5 ± 33.2	< 0.001
Total bilirubin, mg/dL	0.6 ± 0.5	0.7 ± 0.5	0.6 ± 0.4	0.168
Uric acid (mg/dL)	5.8 ± 1.9	5.7 ± 1.8	6.7 ± 2.4	< 0.001
CRP (mg/dL)	0.96 ± 2.62	0.89 ± 2.50	1.89 ± 3.70	< 0.001
Proteinuria, n (%)	538 (17.5)	413 (14.6)	125 (53.0)	< 0.001
PNI	52.1 ± 7.8	52.5 ± 7.5	46.6 ± 9.2	< 0.001
Contrast volume (ml)	243.4 ± 93.1	242.1 ± 90.9	260.9 ± 116.2	0.010

*AKI* acute kidney injury, *BP* blood pressure, *RDW-CV* red cell distribution width coefficient of variation, *eGFR* estimated glomerular filtration rate, *CRP* C-reactive protein, *PNI* prognostic nutritional index.

### Prognostic nutritional index affects the development of contrast associated acute kidney injury

Table 2 lists several factors associated with AKI. On multivariate logistic regression analysis adjusted for age, sex, diabetes mellitus, hypertension, body mass index, hemoglobin, RDW-CV, eGFR, uric acid, CRP, proteinuria, and contrast volume, low PNI was independently associated with an increased risk of AKI (OR 0.96, 95% CI 0.94–0.98, *P* = 0.001).

**Table 2:** Factor related to the development of contrast associated acute kidney injury.

	Univariate analysis		Multivariable analysis	
	OR [95% CI]	*p* value	OR [95% CI]	*p* value
Age	1.03 [1.02, 1.05]	< 0.001	1.00 [0.98, 1.01]	0.800
Sex (female)	1.42 [1.10, 1.84]	0.008	1.34 [0.95, 1.89]	0.099
Diabetes mellitus	2.35 [1.83, 3.01]	< 0.001	1.23 [0.89, 1.72]	0.215
Hypertension	1.73 [1.35, 2.21]	< 0.001	1.05 [0.76, 1.45]	0.772
Current smoker	0.86 [0.66, 1.13]	0.290	–	–
Systolic BP	1.00 [1.00, 1.01]	0.250	–	–
Diastolic BP	1.01 [1.00, 1.02]	0.248	–	–
Body mass index	0.95 [0.91, 0.98]	0. 006	0.99 [0.94, 1.03]	0.576
Hemoglobin	0.73 [0.69, 0.78]	< 0.001	0.98 [0.89, 1.08]	0.707
RDW-CV	1.17 [1.09, 1.26]	< 0.001	1.01 [0.90, 1.13]	0.832
Ln (Total lymphocyte)	0.41 [0.32, 0.53]	< 0.001	–	–
albumin	0.22 [0.18, 0.27]	< 0.001	–	–
eGFR	0.96 [0.96, 0.97]	< 0.001	0.98 [0.97, 0.99]	< 0.001
Total bilirubin	0.75 [0.52, 1.08]	0.127	–	–
Uric acid	1.27 [1.20, 1.35]	< 0.001	1.11 [1.03, 1.19]	0.008
CRP	1.09 [1.06, 1.13]	< 0.001	0.98 [0.94, 1.03]	0.451
Proteinuria	6.60 [5.01, 8.69]	< 0.001	3.24 [2.29, 4.52]	< 0.001
PNI	0.91 [0.89, 0.92]	< 0.001	0.96 [0.94, 0.98]	0.001
Ln (Contrast volume)	1.45 [1.03, 2.04]	0.032	1.60 [1.09, 2.36]	0.018

*OR* odds ratio, *CI* confidence interval, *BP* blood pressure, *RDW-CV* red cell distribution width coefficient of variation, *eGFR* estimated glomerular filtration rate, *CRP* C-reactive protein, *PNI* prognostic nutritional index.

We further divided PNI as a categorical variable. The cut-off level was set to 47.8 based on the ROC curve by AKI prediction (Supplementary Fig. 1). The group with PNI < 47.8 was 1.85 times more susceptible to AKI (Supplementary Table 1) on multiple logistic regression analysis (OR 1.85, 95% CI 1.31–2.64, P = 0.001).

### Both prognostic nutritional index and contrast associated acute kidney injury affect patients’ survival

**Figure 1:**
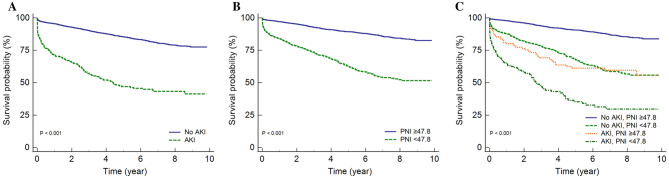
Kaplan–Meier curves for patients’ survival. The survival probability of all-cause mortality in patients (**A**) between patients without AKI and AKI, (**B**) between PNI ≥ 47.8 and PNI < 47.8, (**C**) no AKI/PNI ≥ 47.8, no AKI/PNI < 47.8, AKI/PNI ≥ 47.8, and AKI/PNI < 47.8. PNI, prognostic nutritional index; AKI, acute kidney injury.

The median follow-up duration of the patients was 4.3 years [min 0, max 9.8]. Figure 1 shows the Kaplan–Meier curves for patient survival. The subjects with AKI had higher mortality than those in the no AKI group (*P* < 0.001) (Fig. 1A), and the subjects with lower PNI < 47.8 had higher mortality than those with PNI ≥ 47.8 group (*P* < 0.001) (Fig. 1B). When we divided the groups according to the presence of AKI and PNI, the group with AKI/ PNI < 47.8 had the worst survival (Fig. 1C).

In multiple Cox regression analysis adjusted for age, sex, diabetes mellitus, hypertension, smoking, body mass index, hemoglobin, RDW-CV, eGFR, uric acid, CRP, proteinuria, AKI, medication usage at discharge such as statin and angiotensin II receptor blocker (ARB)/angiotensin-converting enzyme inhibitor (ACEI), low PNI was a significant risk factor for mortality (HR 0.98, CI 0.96–0.99, *P* = 0.003) (Table 3). Age, diabetes mellitus, smoking, body mass index, RDW-CV, CRP, proteinuria, AKI, and usage of ARB/ACEI were also significant factors. The results of multiple Cox regression analysis using PNI as a categorical variable are shown in Supplementary Table 2. Further analysis for the cause of death, such as cardiac death or non-cardiac death, is shown in Supplementary Table 3. Low PNI was still a significant risk factor for cardiac death [HR 0.96, CI 0.93–0.98, *P* = 0.003], and showed similar trend for non-cardiac death [HR 0.98, CI 0.96–1.00, *P* = 0.075].

**Table 3:** Prognostic factors related to patients’ mortality.

	Univariate analysis		Multivariable analysis	
	HR [95% CI]	*p* value	HR [95% CI]	*p* value
Age	1.08 [1.07, 1.09]	< 0.001	1.07 [1.06, 1.09]	< 0.001
Sex (female)	1.28 [1.09, 1.51]	0.003	0.85 [0.68, 1.06]	0.137
Diabetes mellitus	1.75 [1.49, 2.05]	< 0.001	1.52 [1.22, 1.88]	< 0.001
Hypertension	1.27 [1.09, 1.49]	0.002	0.88 [0.72, 1.08]	0. 228
Current smoker	0.77 [0.65, 0.92]	0.003	1.27 [1.00, 1.60]	0.047
Systolic BP	1.00 [0.99, 1.00]	0.367	–	–
Diastolic BP	1.00 [0.99, 1.00]	0.076	–	–
Body mass index	0.85 [0.83, 0.88]	< 0.001	0.93 [0.90, 0.96]	< 0.001
Hemoglobin	0.73 [0.70, 0.76]	< 0.001	0.99 [0.93, 1.05]	0.748
RDW-CV	1.24 [1.20, 1.28]	< 0.001	1.13 [1.07, 1.20]	< 0.001
Ln (Total lymphocyte)	0.39 [0.33, 0.46]	< 0.001	–	–
Albumin	0.25 [0.22, 0.28]	< 0.001	–	–
eGFR	0.97 [0.97, 0.97]	< 0.001	0.99 [0.99, 1.00]	0.051
Total bilirubin	1.16 [1.06, 1.26]	0.001	–	–
Uric acid	1.14 [1.10, 1.19]	< 0.001	1.05 [1.00, 1.11]	0.069
CRP	1.11 [1.10, 1.13]	< 0.001	1.05 [1.02, 1.08]	0.001
Proteinuria	3.31 [2.76, 3.96]	< 0.001	1.48 [1.15, 1.89]	0.002
PNI	0.90 [0.89, 0.91]	< 0.001	0.98 [0.96, 0.99]	0.003
AKI	4.54 [3.796, 5.49]	< 0.001	2.17 [1.66, 2.84]	< 0.001
Statin at discharge	0.68 [0.54, 0.86]	0.001	0.79 [0.61, 1.03]	0.084
ARB/ACEI at discharge	0.61 [0.50, 0.74]	< 0.001	0.65 [0.52, 0.82]	< 0.001

*HR* hazard ratio, *CI* confidence interval, *BP* blood pressure, *RDW-CV* red cell distribution width coefficient of variation, *eGFR* estimated glomerular filtration rate, *CRP* C-reactive protein, *PNI* prognostic nutritional index, *AKI* acute kidney injury, *ARB* angiotensin II receptor blocker, *ACEI* angiotensin converting enzyme inhibitor.

When the patients were divided according to the presence of AKI and classified between PNI < 47.8 or ≥ 47.8, the groups with no AKI/PNI < 47.8 (HR 1.33, CI 1.04–1.69, *P* = 0.022) as well as AKI/PNI < 47.8 (HR 2.55, CI 1.77–3.66, *P* < 0.001) had increased risk of mortality compared to the group with no AKI/PNI ≥ 47.8 (Table 4).

**Table 4:** Cox regression analysis for mortality groups by AKI/PNI.

	Multivariable analysis^a^	
	HR [95% CI]	*p* value
No AKI/PNI ≥ 47.8	Reference	–
No AKI/PNI < 47.8	1.33 [1.04, 1.69]	0.022
AKI/PNI ≥ 47.8	2.76 [1.87, 4.07]	< 0.001
AKI/PNI < 47.8	2.55 [1.77, 3.69]	< 0.001

^a^Adjusted with age, sex, diabetes mellitus, hypertension, smoking, body mass index, hemoglobin, RDW-CV, eGFR, uric acid, CRP, proteinuria, AKI, medication usage at discharge such as statin and angiotensin II receptor blocker (ARB)/angiotensin converting enzyme inhibitor (ACEI).

*HR* hazard ratio, *CI* confidence interval, *AKI* acute kidney injury, *PNI* prognostic nutritional index.

**Figure 2:**
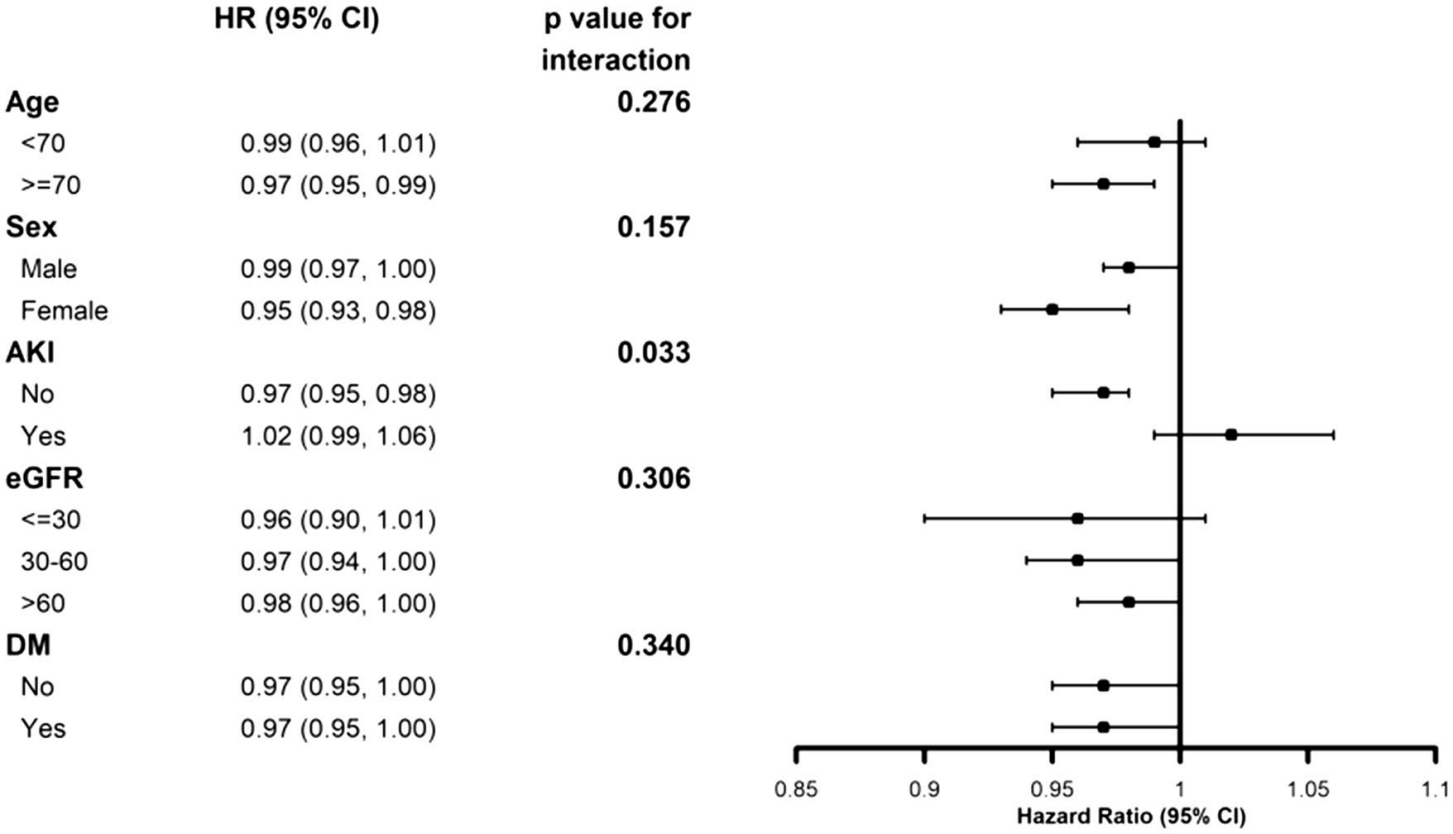
Cox hazard ratio of survival for PNI in various subgroups. A low level of PNI was associated with increased mortality in the group aged over 70 years, female sex, no AKI, and eGFR > 30 ml/min/1.73m^2^. PNI, prognostic nutritional index; AKI, acute kidney injury; eGFR, estimated glomerular filtration rate.

We further analyzed the effects of PNI on various subgroups (Fig. 2). Low PNI levels were associated with increased mortality in the group over 70 years, female sex, no AKI, eGFR > 30 ml/min/1.73m^2^, and with or without diabetes. AKI had interaction with PNI (*P* for interaction = 0.033), which means PNI had different effect on mortality according to AKI or no AKI.

## Discussion

PNI, as a clinical nutritional index, was closely associated with acute kidney outcome and patient mortality after PCI. Low PNI increases the risk of CA-AKI and mortality after PCI. Patients with CA-AKI and low PNI had the highest mortality rates. Low PNI increases mortality, especially in patients over 70 years or female, and is particularly related to cardiac death. Kidney dysfunction and cardiovascular disease are interconnected and can lead to long-term outcomes, such as mortality. The present study was the first to identify the impact of nutrition on CA-AKI and mortality after PCI.

We defined CA-AKI according to the KDIGO guidelines^8^. Traditionally, contrast-induced AKI was defined as a rise in SCr of ≥ 0.5 mg/dL (≥ 44 μmol/L) or a 25% increase from the baseline value occurring two to five days after contrast exposure. However, this definition was limited in that it missed minor increases in creatinine that correlated with adverse outcomes^6^. Furthermore, other factors, such as medication or hypotension, may precipitate AKI after exposure to contrast media, so the term ‘CA-AKI’ is preferred^7^. The incidence of CA-AKI after PCI is varied due to different definitions of CA-AKI and study population characteristics. The incidence of CA-AKI after PCI in this study was 7.3%, and 226 (83.4%) of the cases belonged to the AKI stage I.

We confirmed that a low PNI increased the risk of CA-AKI. Similarly, previous studies have reported the effects of nutrition on AKI. Epidemiology study revealed that patients with NRS-2002 scores ≥ 3 had 1.8 times higher incidence of AKI in a 5-year retrospective cohort study^10^. Experimental studies demonstrated that several nutrients serve as renoprotective agents in AKI. For instance, glutamine administration prevented AKI by downregulating oxidative stress in sepsis-induced AKI rat models^21^. Omega 3 fatty acids decrease polymorphonuclear leukocyte recruitment and cytokine levels in the kidney in ischemic renal injury model^22^. The current study was focused on PNI as a composite nutritional index; PNI is the summation of serum albumin and total lymphocyte counts. Albumin does not only represents the nutritional status^23^ but also acts as an important circulating antioxidant^24^. Albumin acts through multiple binding sites and has free radical-trapping properties. The main mechanisms of contrast-induced nephropathy are vasoconstriction and oxidative stress, followed by renal medullary hypoxia and direct tubular toxicity by contrast media. Thus, a low level of albumin exacerbated CA-AKI. Likewise, lymphocytes can be used as a marker for nutritional status^23^, but various lineages of lymphocytes are also involved in the repair and protection of AKI^25^.

We also revealed several risk factors for CA-AKI. Preexisting CKD, such as low eGFR and proteinuria, is the strongest risk factor for AKI. Serum uric acid is a risk factor for various heart and kidney diseases and was associated with an increased risk of AKI after coronary angiography^12^. The type and volume of the contrast material affected the incidence of AKI. Current guidelines recommend using iso-osmolar or low-osmolar contrast media with minimized volume (total contrast volume/GFR < 3.7) to prevent AKI^26^. All contrast media used in our patients were nonionic iso-osmolar or low-osmolarity media. The mean volume of contrast media injected was 243 mL, and 29.9% of patients received a dose over 3.7 in total contrast volume/GFR. For the high volume of contrast media associated with AKI, we should try to use contrast media cautiously in CKD patients. Diabetes mellitus was not related to the occurrence of AKI.

Various risk factors for mortality after PCI were identified, and AKI showed the highest HR on multiple Cox regression analysis. There is concern that CA-AKI may have been overestimated, affecting the negative effect of performing angiography in patients with CKD^27^. For instance, Ribitsch et al. showed that the occurrence of contrast-induced AKI did not negatively impact kidney function after three months or mortality after PCI in a prospective cohort^28^. However, Ribitsch’s study included only 706 patients with a follow-up period of two years. With a large population and longer follow-up duration, our results clearly showed AKI after PCI as a risk factor for mortality; this is consistent with previous reports^29,30^.

Old age, diabetes mellitus, smoking, and several laboratory values, such as RDW-CV and CRP, were independently associated with increased mortality. CRP, a well-known acute-phase reactant, reflects the degree of underlying inflammation^31^. RDW-CV is a hematological index used to explore the causes of anemia. However, it is now regarded as a prognostic marker that reflects the underlying inflammatory state and is related to patient outcomes^32^. Higher RDW-CV was also a predictor in patients undergoing PCI^33^ in the previous report as well as CRP^34^. Our results are in line with those of previous studies, showing that inflammation played a crucial role in the initiation and progression of atherosclerosis, affecting clinical outcomes.

Meanwhile, a low PNI also increases the risk of mortality after PCI. A previous study also reported that PNI was associated with mortality in patients with stable coronary artery disease^19^. Nutritional influences on cardiovascular risk are well demonstrated. Poor quality of diet and undernutrition could aggravate insulin resistance and atherogenesis, promoting proinflammatory process, eventually leading to mortality^35,36^.

PNI did not show discrimination power for mortality in groups with AKI and eGFR < 30 ml/min/1.73 m^2^. This result could be due to the small population number in AKI (n = 271) groups and eGFR < 30 ml/min/1.73m^2^ (n = 154). Another study reported that malnutrition in AKI patients was associated with a higher incidence of complications, longer hospitalizations, and higher mortality^37^.

We set the cutoff value of PNI as 47.8, defined by ROC curve using AKI prediction. ROC curve for mortality also set a similar cutoff value [data not shown]. However, we could not define a specific PNI cutoff value. After Onodera suggested the cutoff of PNI value as 45^13^, this is frequently used^17,38^. However, the researchers chose several cutoff levels according to study design, such as median, tertile, or ROC analysis^15,18,20,39^. In previous studies, the PNI cut-off value for defining undernutrition varied between 45 and 50. The optimal cut-off value and the classification of the groups by PNI remain unclear.

This study had several limitations. First, this was a single-center retrospective study. Many confounding factors could influence the prognosis of PCI. Second, the PNI was evaluated only once before the PCI. Laboratory changes after PCI were not counted for the analysis. Third, the detection rate of AKI may have been underdiagnosed because of the early discharge of patients. Moreover, we could not suggest AKI to CKD progression or kidney function decline after PCI due to limited follow-up serum creatinine data. We did not consider heart failure; heart failure is one of the important factors for AKI and mortality. In addition, AKI and PNI are highly correlated. To solve the multicollinearity problem, we categorized and integrated AKI/PNI variables. Finally, we did not perform another nutritional assessment, such as subjective global assessment or body composition. However, we analyzed a large number of patients and analyzed long-term patient outcomes using national statistical data related to death.

In conclusion, poor nutritional status, as assessed by PNI, was a risk factor for CA-AKI and all-cause mortality in patients undergoing PCI. This finding suggested that the PNI score, which can be easily determined using serum albumin and lymphocyte counts, was a useful prognostic marker in patients undergoing PCI. Clinicians should be careful when diagnosing and treating patients with coronary artery disease with low PNI.

## Methods

### Study population

We retrospectively screened 4,096 patients who underwent PCI at Pusan National University Hospital between January 2010 and December 2018. If the subjects underwent repetitive PCI during the study period, only the first case was included. Patients who already received hemodialysis due to kidney failure (n = 137), died within 48 h after PCI (n = 52) or did not have serum creatinine (n = 45) or PNI (n = 131) values, were excluded. A total of 3,731 subjects were included in the final analysis.

### Data sources and measures

The data were retrieved from electronic medical records. The data collected included age, sex, body mass index, history of diabetes, hypertension, blood pressure, and laboratory findings, including serum creatinine, hemoglobin, albumin, and urine dipstick albumin results. Mortality data were obtained from the Microdata Integrated System (MDIS)^8^, generated by Statistics Korea.

CA-AKI was defined as a rise in serum creatinine ≥ 0.3-or 1.5 than the baseline value within 48 h after PCI according to KDIGO guidelines^40^. Estimated GFR (eGFR) was calculated through the CKD-EPI equation^41^. Proteinuria was defined as a urine dipstick test result of ≥ 1 +^42^. PNI was calculated as 10 × serum albumin (g/dL) + 0.005 × total lymphocyte count (per mm^3^)^13^.

### Statistical analysis

All variables with a normal distribution were expressed as mean ± standard deviation. Categorical variables were expressed as numbers and proportions, and comparisons were made using the chi-square test. Logistic regression analysis was conducted to analyze the factors associated with AKI. The ability to predict CA-AKI was evaluated using the area under the curve (AUC) in the receiver operating characteristic (ROC) curve. The cumulative survival rates were estimated using the Kaplan–Meier method, and differences between survival curves were compared using the log-rank test. Multivariate Cox regression models for mortality were constructed using the confounding factors. Significant variables in the univariate model were entered into a multivariable model. Among the variables collected, serum albumin levels and lymphocyte counts were not included in the multiple logistic or Cox regression models because they were included in the calculation of PNI. Variables included in all multivariable analyses were tested for multicollinearity. We also examined the association between PNI and mortality through a subgroup analysis using a fully adjusted multivariate Cox regression model. The patients were stratified according to age, sex, presence of diabetes mellitus, baseline eGFR, and CA-AKI. All analyses were performed using the R statistical software package (version 4.0.1; R Development Core Team; R Foundation for Statistical Computing, Vienna, Austria).

### Ethical statement

The study was conducted in accordance with the Declaration of Helsinki, and the study was approved by the Institutional Review Board of Pusan National University Hospital (H-1909-014-083). The need for informed consent was waived because of its retrospective design.

## Supplementary Information

Supplementary Figure and Tables.
