# Supplementary material for: Impact of nutritional index on contrast-associated acute kidney injury and mortality after percutaneous coronary intervention
Source: Sci Rep. 2021 Mar 29;11:7123. doi: 10.1038/s41598-021-86680-7 (PMC8007688; doi:10.1038/s41598-021-86680-7)
Supplement: Supplementary file 1 — Supplementary Figure and Tables. [file 41598_2021_86680_MOESM1_ESM.docx]

Supplementary Figure 1. Receiver operating characteristics (ROC) analysis of PNI for acute kidney injury.

The area under the curve for PNI is 0.707 and the cut off value is 47.8.


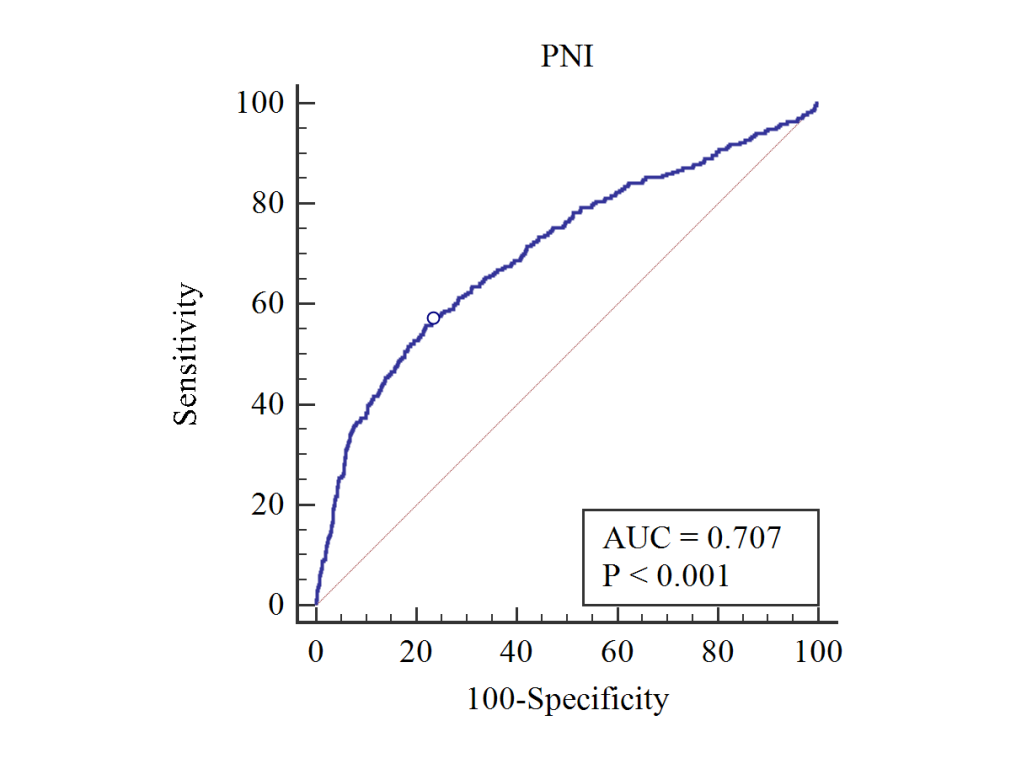


Supplementary Table 1. Multivariate logistic analysis for variables related to acute kidney injury, PNI as a categorical variable

|  | Multivariable analysis | | |
| --- | --- | --- | --- |
|  |  | OR [95% CI] | p-value |
| age |  | 1.00 [0.98, 1.02] | 0.910 |
| Sex (female) |  | 1.38 [0.97, 1.96] | 0.076 |
| Diabetes mellitus |  | 1.21 [0.87, 1.70] | 0.254 |
| Hypertension |  | 1.06 [0.77, 1.46] | 0.727 |
| Body mass index |  | 0.99 [0.94, 1.03] | 0.555 |
| current smoker |  | 1.15 [0.81, 1.65] | 0.438 |
| Hemoglobin |  | 0.96 [0.88, 1.06] | 0.443 |
| RDW-CV |  | 1.01 [0.91, 1.13] | 0.87 |
| eGFR |  | 0.98 [0.97, 0.99] | <0.001 |
| Uric acid |  | 1.11 [1.03, 1.19] | 0.009 |
| CRP |  | 0.99 [0.95, 1.03] | 0.553 |
| proteinuria |  | 3.14 [2.23, 4.42] | <0.001 |
| PNI < 47.8 |  | 1.85 [1.31, 2.64] | 0.001 |
| Ln (Contrast volume) |  | 1.59 [1.08, 2.35] | 0.019 |

OR, odds ratio; CI, confidence interval; RDW-CV, red cell distribution width coefficient of variation; eGFR, estimated glomerular filtration rate; CRP, C-reactive protein; PNI, prognostic nutritional index

Supplementary Table 2. Multivariate Cox regression analysis for mortality, PNI as a categorical variable

|  | Multivariable analysis | |  |
| --- | --- | --- | --- |
| Variables | HR [95% CI] | p-value | |
| Age | 1.07 [1.06, 1.09] | <0.001 | |
| Sex (female) | 0.82 [0.66, 1.02] | 0.074 | |
| Diabetes mellitus | 1.50 [1.21, 1.87] | <0.001 | |
| Hypertension | 0.89 [0.72, 1.09] | 0.253 | |
| Current smoker | 1.23 [0.97, 1.55] | 0.083 | |
| Body mass index | 0.93 [0.90, 0.96] | <0.001 | |
| PNI < 47.8 | 1.25 [1.00, 1.58] | 0.053 | |
| Hemoglobin | 0.98 [0.92, 1.04] | 0.425 | |
| RDW-CV | 1.13 [1.07, 1.20] | <0.001 | |
| eGFR | 0.99 [0.99, 1.00] | 0.051 | |
| Uric acid | 1.05 [0.99, 1.10] | 0.097 | |
| CRP | 1.05 [1.03, 1.08] | <0.001 | |
| proteinuria | 1.52 [1.19, 1.95] | 0.001 | |
| AKI | 2.23 [1.70, 2.91] | <0.001 | |
| Statin at discharge | 0.79 [0.60, 1.03] | 0.076 | |
| ARB/ACEI at discharge | 0.66 [0.53, 0.83] | <0.001 | |

HR, hazard ratio; CI, confidence interval; RDW-CV, red cell distribution width coefficient of variation; eGFR, estimated glomerular filtration rate; CRP, C-reactive protein; PNI, prognostic nutritional index; AKI, acute kidney injury; ARB, angiotensin II receptor blocker; ACEI, angiotensin converting enzyme inhibitor

Supplementary Table 3. Multivariate Cox regression analysis for mortality according to cause of death

|  | Cardiac death | | | Non-cardiac death | | |
| --- | --- | --- | --- | --- | --- | --- |
|  | HR [95% CI] | p-value |  | | HR [95% CI] | p-value |
| Age | 1.06 [1.03-1.08] | <0.001 |  | | 1.08 [1.07-1.10] | <0.001 |
| Sex (female) | 1.00 [0.66-1.50] | 0.979 |  | | 0.82 [0.63-1.07] | 0.147 |
| Diabetes mellitus | 1.54 [1.04, 2.30] | 0.032 |  | | 1.50 [1.16-1.95] | 0.002 |
| Hypertension | 0.80 [0.55, 1.16] | 0.236 |  | | 0.91 [0.71-1.17] | 0.462 |
| Current smoker | 1.38 [0.92, 2.07] | 0.123 |  | | 1.22 [0.92-1.62] | 0.161 |
| Body mass index | 0.93 [0.87, 0.98] | 0.007 |  | | 0.92 [0.89-0.95] | <0.001 |
| Hemoglobin | 1.05 [0.94, 1.18] | 0.388 |  | | 0.97 [0.90-1.05] | 0.440 |
| RDW-CV | 1.13 [1.01, 1.27] | 0.034 |  | | 1.14 [1.06-1.22] | <0.001 |
| eGFR | 0.99 [0.98, 1.00] | 0.046 |  | | 1.00 [0.99-1.00] | 0.169 |
| Uric acid | 1.03 [0.93, 1.13] | 0.607 |  | | 1.06 [1.00-1.13] | 0.067 |
| CRP | 1.07 [1.03, 1.11] | 0.001 |  | | 1.05 [1.01-1.09] | 0.012 |
| proteinuria | 1.68 [1.09, 2.59] | 0.019 |  | | 1.51 [1.12, 2.04] | 0.007 |
| PNI | 0.96 [0.93, 0.98] | 0.003 |  | | 0.98 [0.96-1.00] | 0.075 |
| AKI | 1.61 [0.94, 2.74] | 0.083 |  | | 2.59 [1.90-3.54] | <0.001 |
| Statin at discharge | 0.80 [0.49. 1.30] | 0.367 |  | | 0.71 [0.52-0.98] | 0.038 |
| ARB/ACEI at discharge | 0.55 [0.37, 0.82] | 0.003 |  | | 0.70 [0.53-0.92] | 0.010 |

HR, hazard ratio; CI, confidence interval; RDW-CV, red cell distribution width coefficient of variation; eGFR, estimated glomerular filtration rate; CRP, C-reactive protein; PNI, prognostic nutritional index; AKI, acute kidney injury; ARB, angiotensin II receptor blocker; ACEI, angiotensin converting enzyme inhibitor
